# Phase retrieval based on deep learning in grating interferometer

**DOI:** 10.1038/s41598-022-10551-y

**Published:** 2022-04-25

**Authors:** Ohsung Oh, Youngju Kim, Daeseung Kim, Daniel. S. Hussey, Seung Wook Lee

**Affiliations:** 1 School of Mechanical Engineering, Pusan National University, Busan, 46241 Republic of Korea; 2 Department of Chemistry and Biochemistry, University of Maryland, College Park, MD 20742 USA; 3 Neutron Physics Group, National Institute of Standards and Technology, Gaithersburg, MD 20899 USA

**Keywords:** Engineering, Imaging and sensing, Imaging techniques

## Abstract

Grating interferometry is a promising technique to obtain differential phase contrast images with illumination source of low intrinsic transverse coherence. However, retrieving the phase contrast image from the differential phase contrast image is difficult due to the accumulated noise and artifacts from the differential phase contrast image (DPCI) reconstruction. In this paper, we implemented a deep learning-based phase retrieval method to suppress these artifacts. Conventional deep learning based denoising requires noise/clean image pair, but it is not feasible to obtain sufficient number of clean images for grating interferometry. In this paper, we apply a recently developed neural network called Noise2Noise (N2N) that uses noise/noise image pairs for training. We obtained many DPCIs through combination of phase stepping images, and these were used as input/target pairs for N2N training. The application of the N2N network to simulated and measured DPCI showed that the phase contrast images were retrieved with strongly suppressed phase retrieval artifacts. These results can be used in grating interferometer applications which uses phase stepping method.

## Introduction

Lab-based X-ray imaging is a widely employed, non-destructive imaging modality finding various applications in clinical and biological areas^1–3^. Conventional transmission attenuation imaging provides limited information about an object. X-ray grating interferometer is a method to obtain more information about the object effectively. This method can use not only synchrotron sources^2,4^, but also conventional X-ray sources^5,6^, neutron sources^7–11^, etc. We obtain three types of images through X-ray grating interferometer which include the transmission, the phase contrast, and dark-field images using phase stepping method^12^. The interactions between the X-ray and the object are described as the complex refractive index n ($=1-\delta +i\beta$), where the imaginary part $\beta$ is related to the absorption and the real part $\delta$ is related to the phase shift of the objects. In the low energy region, $\delta$ is about 1,000 times larger than $\beta$ for low Z materials, such as soft tissues, polymers, etc^1,5,13^. Therefore, the phase information is more sensitive and provides higher contrast than the absorption information in soft materials. However, the phase contrast image using grating interferometer is not phase contrast image (PCI), but it is one-dimensionally differentiated. The differential phase contrast image (DPCI) is strong in edge position, but it requires retrieving phase contrast images for quantitative analysis.

**Figure 1:**
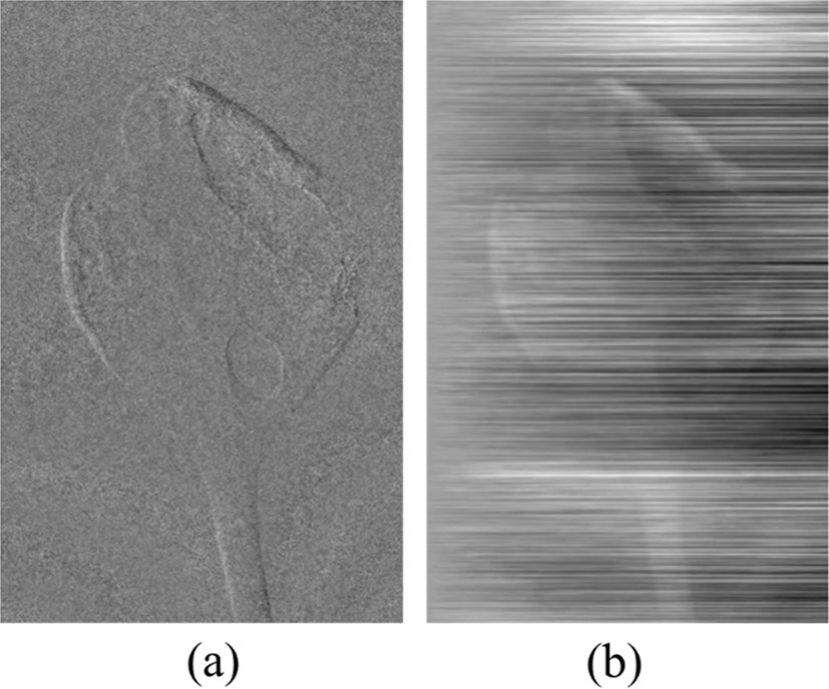
The phase images from grating interferometer. (**a**) The differential phase contrast image, (**b**) the retrieved phase image using direct integration.

There are several methods for retrieving phase information, such as direct integration, filtering^14^, two-directional method^15,16^, and non-linear regularization method^17,18^, etc. Direct integration (shown in Fig. 1) is the simplest method to phase retrieval, but it generates severe artifacts in the PCI, so one cannot use this method directly. Filtering methods remove stripe artifacts using filter functions in the PCIs^14^. These methods are also fast and simple, but the resulting images are blurry, of poor visual image quality, and often contain systematic noise. The two-directional method uses two DPCI images which are obtained along two-orthogonal directions^15,16^. This method can yield high quality images compared to other methods, but it needs additional DPCI image. It requires additional scan time, and it poses the dose problem and the grating alignment problem compared to one-directional method. The non-linear regularization method, which is briefly described in the Methods section below, is retrieving DPCI using an iterative method^17^. This method uses an appropriate cost function to reduce the stripe artifacts and the DPCI is retrieved by minimizing it. It shows better results than filtering methods, but it often introduces image artifacts.

Deep learning methods are promising modeling techniques^19–28^ due to their accuracy enabled by the ongoing improvement of graphical processing unit (GPU) hardware. These methods have been developed for many computer vision tasks, such as image classification^21,22^, segmentation^23,24^, artifact removal^25,26^, denoising^27,28^, etc. In a published study, a deep learning technique for phase retrieval using the filtered image as an input and the reference image as a target was used^29^. Most deep learning methods are data-driven, and require preparation of training data, for instance, noise/clean image pairs for denoising algorithms. However, it is often hard or impossible to prepare noise-free real data, therefore it is impractical for real applications. Recently, the Noise2Noise (N2N) technique have been developed for denoising, which uses noise/noise image pairs, and we adapted this technique for phase retrieval in grating interferometry^30,31^. Grating interferometry uses phase stepping of a moiré pattern and subsequent fitting to obtain the transmission, DPCI and dark-field images, with about 10 raw phase step images typically acquired. We used these phase stepping images to obtain a lot of noisy DPCIs by combination. The proposed N2N network consists of a dual stream. The base stream uses the transmission and the dark-field images as an input and the detail stream uses the DPCIs as an input. This architecture produces a phase contrast image with reduced noise and artifacts and reduces spatial blurring.

## Results

**Figure 2:**
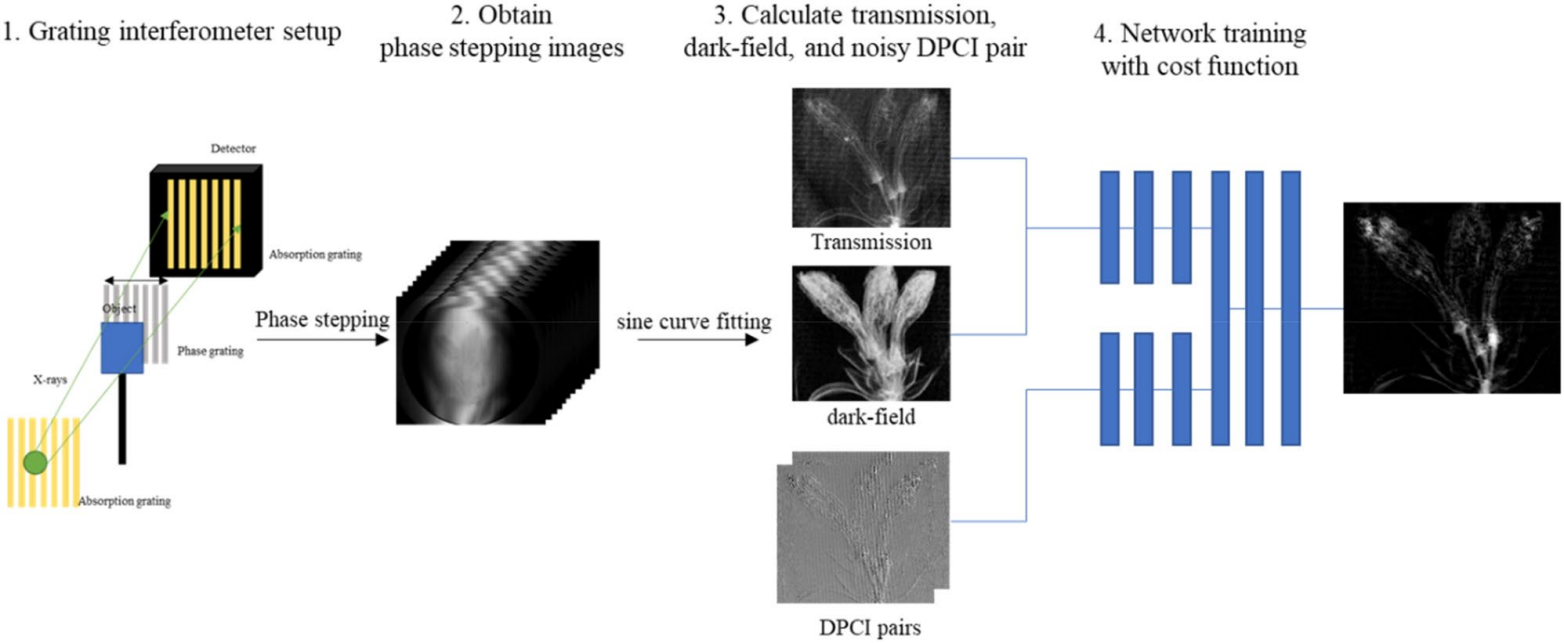
Schematic of deep learning-based phase retrieval in grating interferometer.

**Figure 3:**
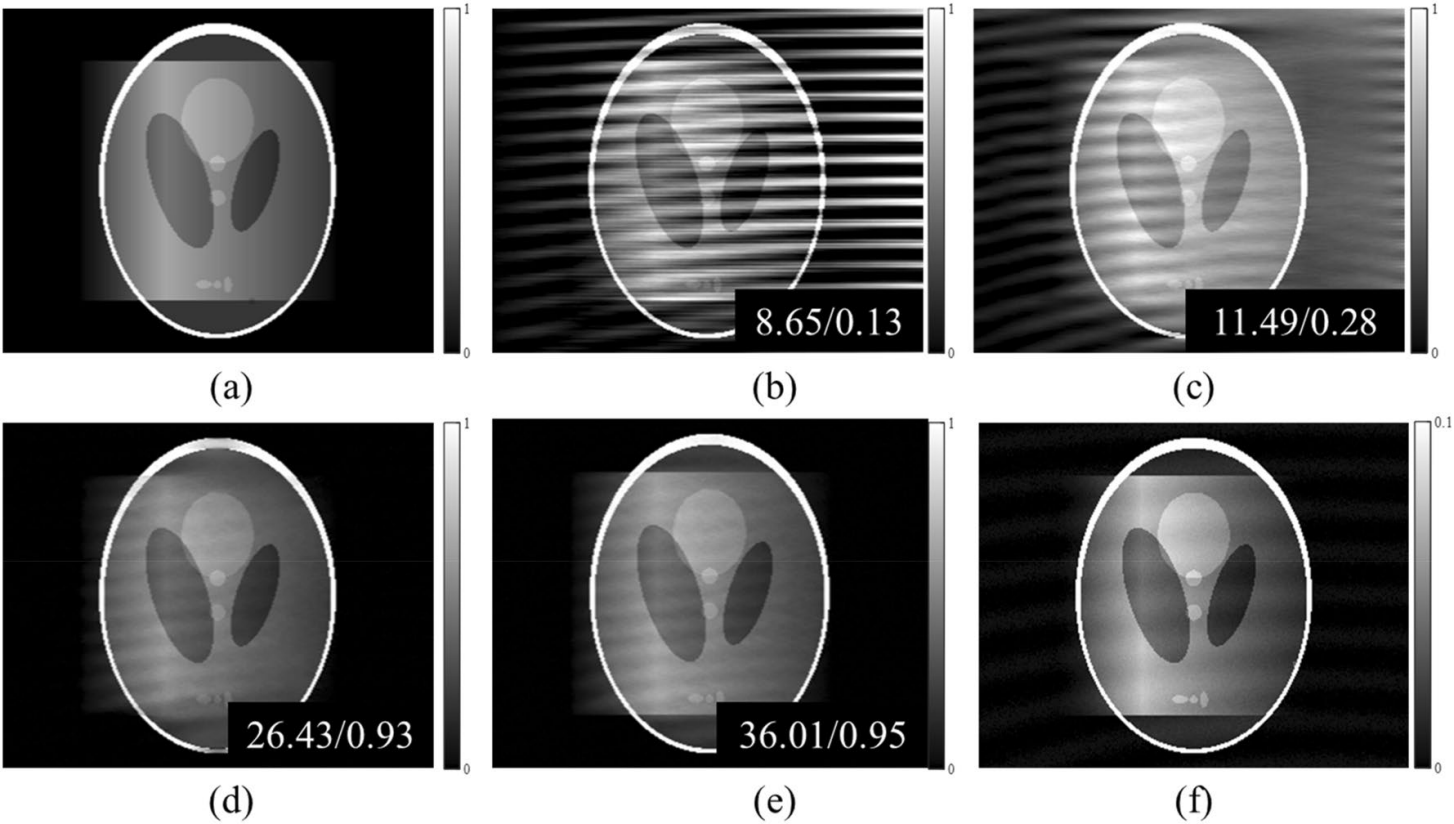
Comparison results for numerical phantom image. (**a**) Reference, (**b**) direct integration, (**c**) Wavelet-Fourier filtering, (**d**) iterative method, (**e**) The proposed method, (**f**) transmission. The display window for (**f**) is [0, 0.1], the others is [0, 1], respectively. The PSNR and SSIM values are displayed in the images.

**Figure 4:**
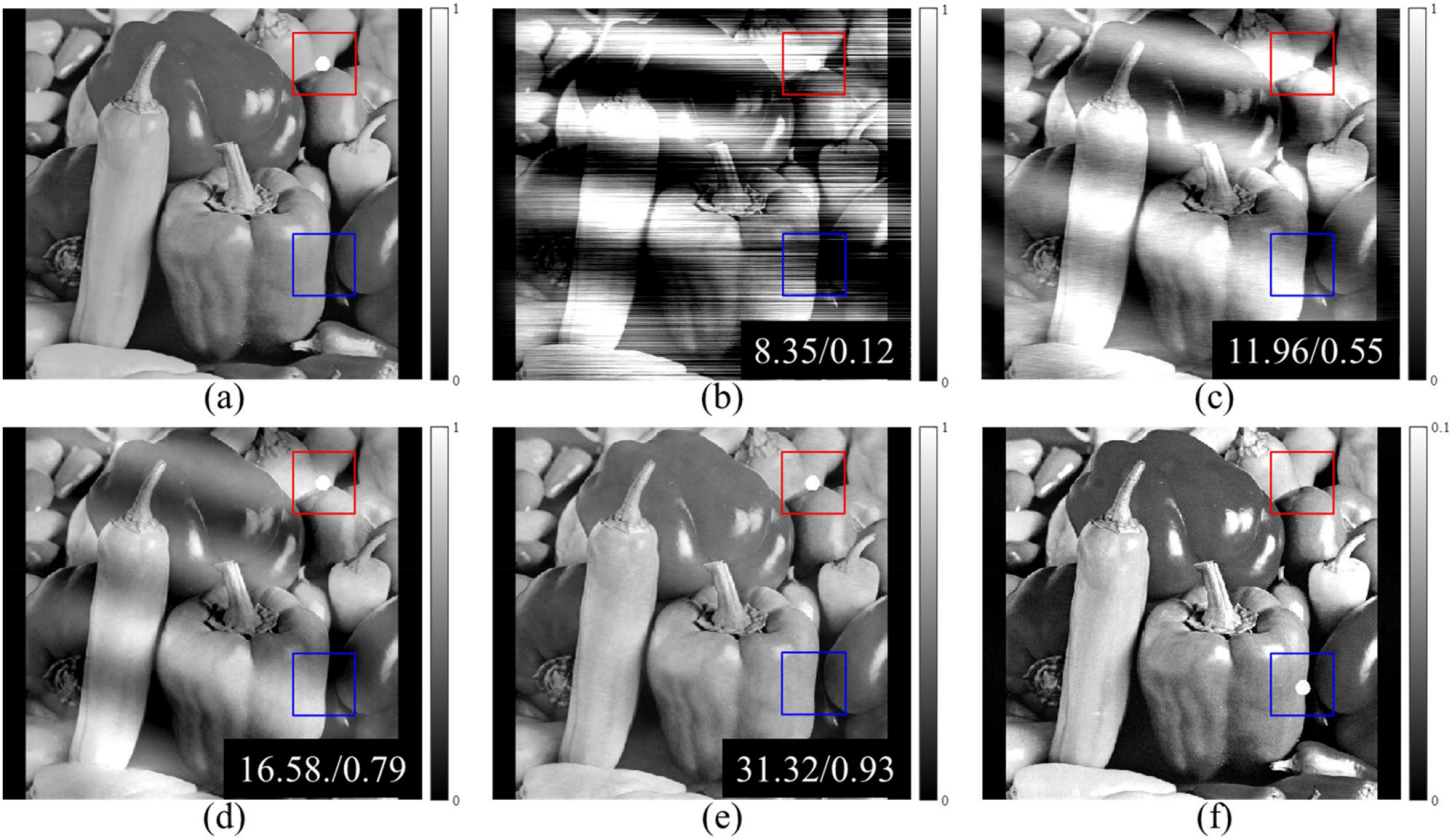
Comparison results for peppers image from USC-SIPI image database. (**a**) Reference, (**b**) direct integration, (**c**) Wavelet-Fourier filtering, (**d**) iterative method, (**e**) The proposed method, (**f**) transmission. The display window for (**f**) is [0, 0.1], the others is [0, 1], respectively. The PSNR and SSIM values are displayed in the images. The blue and red area show the points in the transmission image and the phase contrast image, respectively.

The schematic of the proposed method is shown in Fig. 2. We obtained a set of phase stepping images with and without samples using a grating interferometer with a sinusoidal model of the signal shape. The numerical phantom was created for simulation, and we obtained phase stepping images, using the same signal model. Training of the N2N model requires many noisy images with the same expected values. We obtained the noisy DPCIs using a combination of the phase stepping images, and these images were used as network inputs and targets. The results of the Shepp-Logan phantom obtained from MATLAB are shown in Fig. 3. We compared the resulting image to the results obtained by direct integration, wavelet-Fourier (WF) filtering^14^, and an iterative method^32^. The peak-to-noise ratio (PSNR) and the structure similarity (SSIM) index are displayed in the results. There are several types of noises and artifacts in the DPCIs, including background noise and a moiré artifact due to interferometer alignment error. The direct integration result shows that these noise sources accumulate and produce very poor image quality. WF filtering can remove the horizontally accumulated artifacts, however, it is limited to removing only one directional noise, and hence the result is also of poor visual image quality. The iterative method minimizes a cost function to retrieve phase information, and it yields high quality of the retrieved phase information except from horizontally blurry regions. This is because the DPCI we obtain is a one-directionally differentiated image. Therefore, the horizontal line was not retrieved well. The proposed method achieved better results compared to other methods, with even preserving the horizontal features. The network used in this study leveraged not only DPCIs, but also transmission and dark-field images. The transmission and dark-field images can help to preserve shapes of the phase information. The quantitative analysis showed that the result using the deep learning method is of the highest visual quality. We also used the “peppers image” from USC-SIPI image database which is known for many high frequency features and more complex shapes than the Shepp-Logan phantom. The results presented in Fig. 4 show that the phase information is well retrieved even in the case of a complex scene. The area in the blue box has features shown only in the transmission image, and that in the red box only in the PCI. The results show that the feature in the red box uniquely exiting in the PCI is well preserved in the proposed method while that in the blue box uniquely existing the transmission image has little effect the retrieved PCI. In these results, the N2N network can be employed on any grating interferometry dataset.

**Figure 5:**
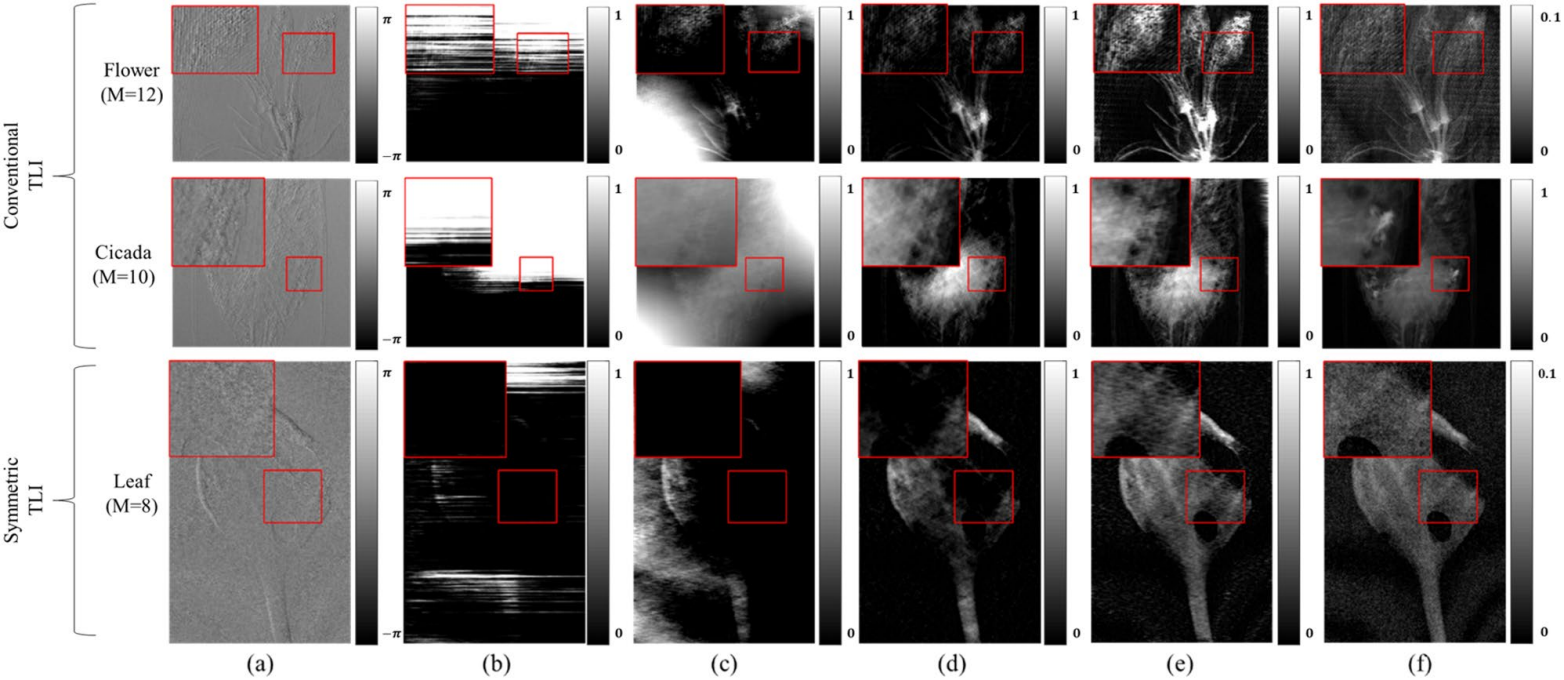
The X-ray grating interferometer comparison results. (**a**) DPCI, (**b**) direct integration, (**c**) WF filtering, (**d**) iterative method, (**e**) the proposed method, (**f**), transmission. The display windows are same for (**b**–**e**), and the display window of the first and third images of (**f**) are [0, 0.1], and the second is [0, 1], respectively. The variable M denotes the total phase stepping number. The red rectangle regions are selected ROIs.

For measured data, we employed images acquired from two separate X-ray grating interferometers and one neutron grating interferometer. The first X-ray grating interferometer is a conventional Talbot-Lau interferometer (TLI) in which G_1_ is close to G_2_ rather than G_0_^3,15^. The second X-ray TLI employed the symmetric geometry in which G_1_ is positioned to middle of the system^8,10,33^. A conventional TLI has the advantage to obtain high spatial resolution images, and symmetric TLI has the advantage to obtain highly sensitivity DPCI^34^. Training datasets were created through random combinations of half of the phase step images for each sample. Figure 5 shows the X-ray TLI results of a flower, a cicada, a leaf, with the first and second row obtained using the conventional geometry, and the third row is obtained from the symmetric geometry. Direct integration method results in very poor image quality, with no discernable detail in the PCI. The WF filtering technique also results in very low PCI quality. The iterative method yields a better PCI than the direct integration and WF filtering, where there are many more discernable features. However, the proposed method can provide significantly better estimates of the phase information compared to other retrieving methods. The resulting PCI has a similar shape as the transmission image, but it has different contrast and details. Therefore, it helps to obtain various information about the objects.

**Figure 6:**
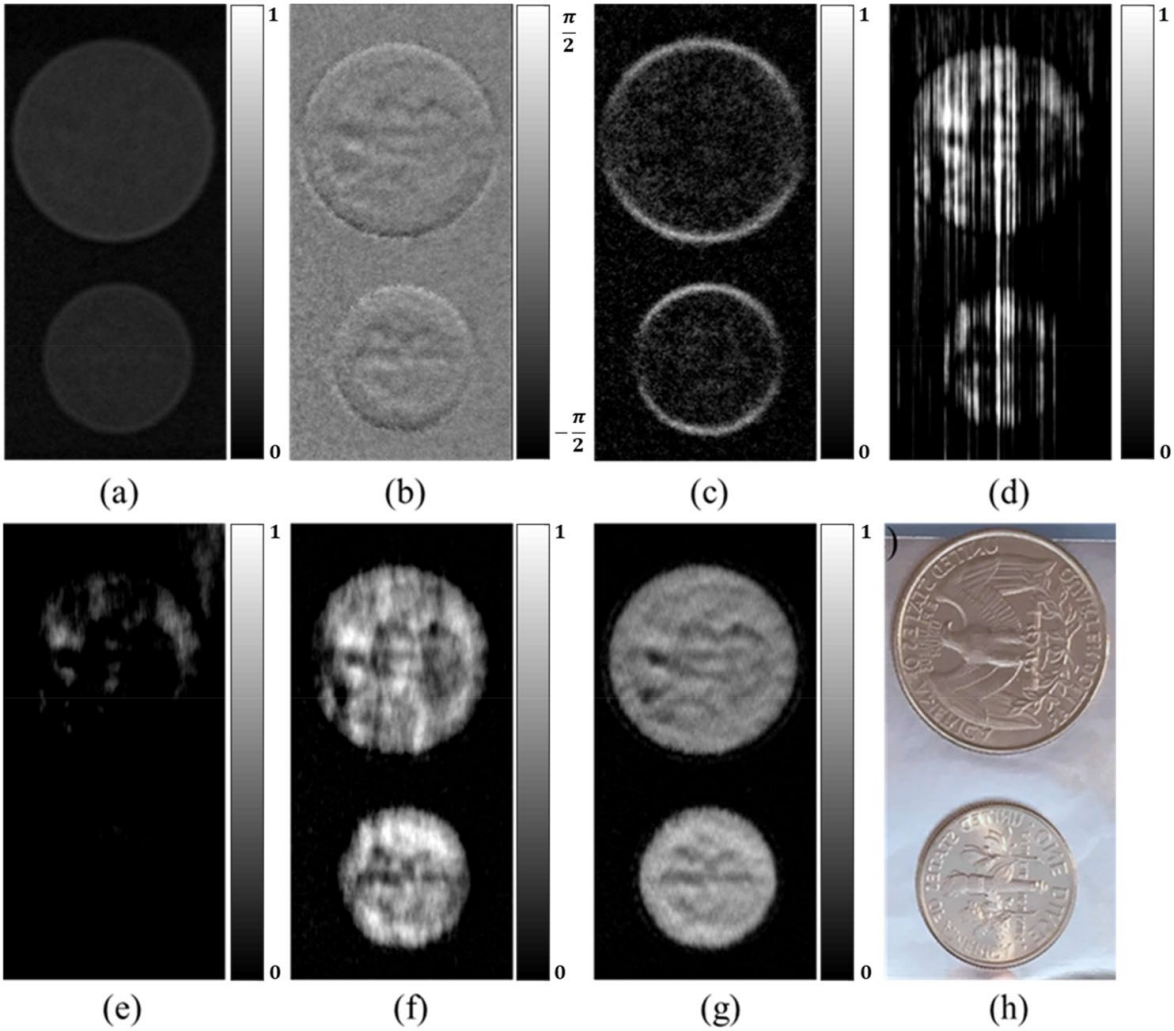
Comparison of PCI retrieval from neutron TLI images of coins. (**a**) Transmission, (**b**) DPCI, (**c**) dark-field, (**d**) direct integration, (**e**) WF filtering, (**f**) iterative method, (**g**) the proposed method, (**h**) visual image. The display windows are same for (**d**–**g**). The total phase stepping number is eight.

**Figure 7:**
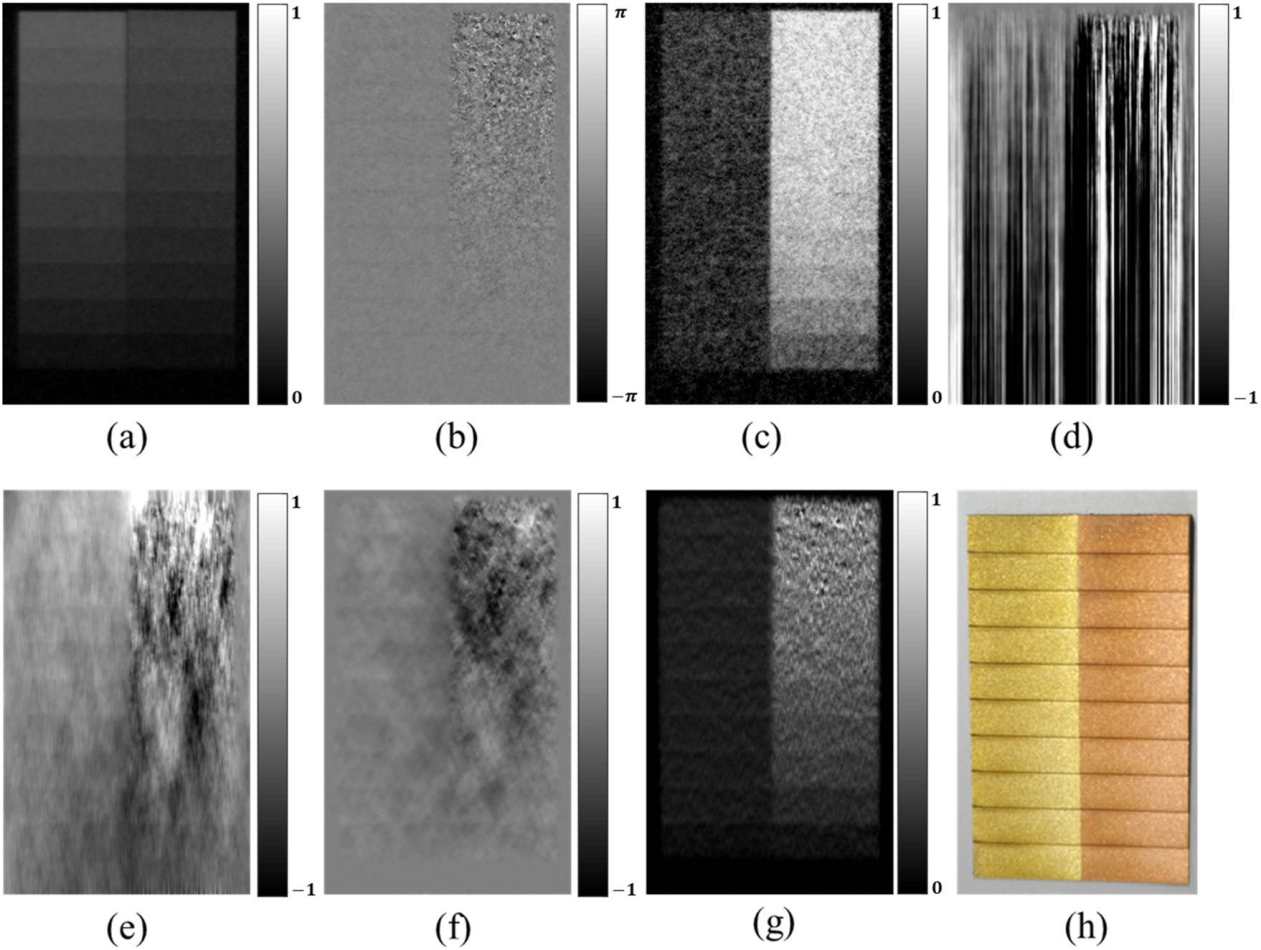
Comparison of PCI retrieval from neutron TLI images of brass and copper steps. (**a**) transmission, (**b**) DPCI, (**c**) dark-field, (**d**) direct integration, (**e**) WF filtering, (**f**) iterative method, (**g**) the proposed method, (**h**) visual image. The display window is [− 1, 1] for (**d**), (**e**), (**f**), and [0, 1] for (**g**). The total phase stepping number is eight.

We also analyzed data from a neutron grating interferometer setup. The samples were coins and a step sample composed of brass and copper as shown in Figs. 6h and 7h. Usually, neutron images are noisier than X-ray images due to the low flux of neutron. Figures 6 and 7 compare the results of the various phase retrieval methods. Similar to the X-ray TLI results, the proposed method produces superior image quality and PCI estimates.

**Figure 8:**
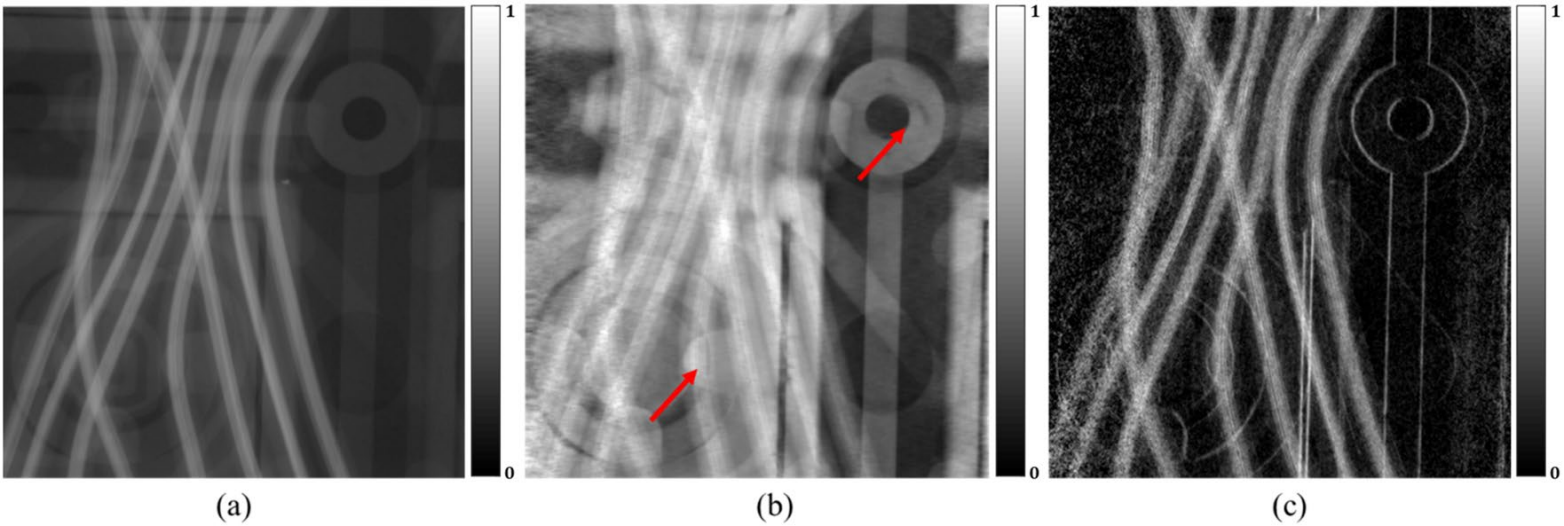
The grating interferometer results of the circuit sample. (**a**) Transmission, (**b**) The retrieved phase, (**c**) the dark-field. Red arrows denote missing information which cannot see in the transmission image. The phase information was retrieved with quite good quality even though there is no prior information of air region.

Lastly, we analyze X-ray TLI images of a circuit board. In this case, the circuit board is larger than the field-of-view (FOV). When analyzing an object that is larger than the TLI FOV, it is not possible to obtain the boundary condition of zero phase, and we don’t set the air region, and the PCI may have low image quality. Figure 8 shows the retrieved PCI of the circuit board by the proposed method. The retrieved phase is of good quality even though mask images don’t exist. The good quality is attributed to the fact that transmission and the dark-field images used as the inputs of the network have similar shapes to the phase image.

## Discussion

The phase retrieval in grating interferometer is one of the important parts for material analysis. However, its use is limited for many reasons, such as motor control error, non-uniform phase coefficient, etc. which often generate severe image artifacts. In this study, we implemented the deep learning method to retrieve the phase contrast image produced by grating interferometers. We showed that this method has several advantages compared other commonly employed phase retrieval methods. First, one can obtain a high-quality PCI with fast acquisition time if the network is previously trained. Second, the N2N deep learning network does not require pseudo-images for training since the N2N does not require clean images and can operate on real data directly. The proposed method can use noisy DPCIs using combination of the phase stepping images, and it is possible to train networks which are fitted for real data. However, there are some limitations to this method. First, the noise and artifacts in the images are not perfectly removed, and some percentage of them still remain in the images. In the published paper^31^, the condition of using N2N is that the dataset is sufficiently large and the conditional expectation between paired noisy images is zero. It means that the noises between each image pair should be zero-mean and independent of each other. In practice, the images used in this paper do not satisfy this condition. However, this condition can be approximately satisfied when the phase stepping number is very large. But due to the radiation dose problem, the value of “very large” and the amount of allowed radiation form a trade-off relationship. Second, we should set mask image before training. The mask images in this paper were obtained manually using threshold to the images. Nonetheless, the manual threshold method can be replaced by advanced and automatic methods for creating mask images.

## Conclusion

The grating interferometer can obtain the information about the objects through various types of the images, and it is important to analyze the information in the images using proper methods. In this study, we implemented a deep learning-based phase retrieval method for grating interferometry. This method can be used both with simulation and measured data; it produces better image quality than conventional methods and has the advantage of being applicable to any grating interferometer employing the phase stepping method regardless of the radiation sources such as X-ray and neutron. We believe that this method will be an effective tool to retrieve the PCI from the DPCI, and it will improve the analysis of complex objects.

## Methods

### Talbot–Lau interferometer

The regular TLI uses three gratings: a source grating G_0_, a phase grating G_1_, an analyzer grating G_2_. The TLI uses diffraction that occurs when X-ray beam passes through the G_1_, and this diffraction pattern is repeated with certain distance by Talbot effect. However, the intensity pattern is usually smaller than the detector pixel size, the G_2_ grating is used to make moiré fringe. This pattern is periodically changed with the G_1_ is moving a its period, and these changes are recorded called phase stepping method. These phase stepping images which changes intensity periodically are represented as sine curve that are described by

1 $${I}_{ref}={a}_{0,ref}+{a}_{1,ref}\mathrm{sin}\left(\frac{2\pi (k+{\eta }_{k,ref})}{M}+{\theta }_{ref}\right)$$

2 $${I}_{sam}={a}_{0,sam}+{a}_{1,sam}\mathrm{sin}\left(\frac{2\pi (k+{\eta }_{k,sam})}{M}+{\theta }_{sam}\right)$$

where ${I}_{ref},{I}_{sam}$ denote the intensity without and with samples, and *a*_*0*_*, a*_*1,*_ $\theta$ denote DC offset, amplitude, and the phase coefficient, respectively. M is a total phase stepping number, and *k* = *1*, *2*, …, *M*. ${\eta }_{k}$ is a mechanical error term that occurs the grating is moved to perform phase stepping. And we used least-square method^35^ for sine curve fitting shown as

3 $$\underset{{a}_{0},{a}_{1},\theta }{\mathrm{min}}\sum_{k=1}^{M}{\Vert {I}_{k}({a}_{0},{a}_{1},\theta )-\widehat{{I}_{k}}\Vert }_{2}^{2}$$

where $I$ and $\widehat{I}$ denote the fitted images and the measured images, respectively. The transmission, DPCI, dark-field images can be calculated from the fitted parameters as follows:

4 $$Transmission=\frac{{a}_{0,sam}}{{a}_{0,ref}}$$

5 $$DPCI= {\theta }_{sam}-{\theta }_{ref}$$

6 $$Dark-field=\frac{{a}_{1,sam}/{a}_{0,ref}}{{a}_{0,sam}/{a}_{1,ref}}$$

### Phase retrieval method

While the transmission image and the dark-field image can be obtained directly from the phase stepping images, however, the PCI we can obtain is one-dimensionally differentiated. The mathematical expression of phase retrieval is shown as

7 $$\phi \left(x, y\right)=c\int\limits_{0}^{x} {\left\{\varphi \left({x}^{^{\prime}},y\right)+n({x}^{^{\prime}},y)\right\}dx {^{\prime}}} $$

8 $${\phi \left(x, y\right)}_{ideal}= c\int\limits_{0}^{x} {\varphi \left({x}^{^{\prime}},y\right)dx {^{\prime}}}$$

where $\phi$ and $\varphi$ denote a PCI and a DPCI, respectively, and *c* is a constant given specification of grating interferometer system, n denotes the noise. If the DPCI is noise-free, the PCI is retrieved using simple one-dimensional integration and there are no noises in the PCI. However, in most cases, there are moiré artifacts due to the motion of the grating and noises in DPCIs, and these are accumulated during integration. Therefore, we do not use one-dimensional integration directly, and need to optimize the images using other methods.

9 $$\underset{x}{\mathrm{min}}{\Vert {D}_{x}{\widehat{x}}_{i}-\varphi \Vert }_{2}^{2}+w$$

where *D* denotes a one-dimensional first-order derivative operator, and *D*_*x*_ is a derivative operator in horizontal direction. And *w* is the regularization term. This cost function can be reformulated as this unconstrained form^17^.

10 $$argmin\left({\Vert {D}_{x}{\widehat{x}}_{i}-\varphi \Vert }_{2}^{2}+{\lambda }_{1}{\Vert TV{\widehat{x}}_{i}\Vert }_{1}^{1}+{\lambda }_{2}{\Vert M{\widehat{x}}_{i}\Vert }_{2}^{2}\right)$$

where *TV* is total variation operator of the images, and M is a mask function that denotes prior information for air region. The first term of regularization term is total variation (TV) term, which is for denoising in PCIs, and the second term is binary term, which is for DC offset calibration. The selection of these parameters is critical for image quality. In this study, we manually set the mask images using threshold to the transmission, DPCI, dark-field images. And we used alternating direction method of multipliers (ADMM) for phase retrieval with ${\lambda }_{1}$ = 0.001 and ${\lambda }_{2}$ = 1, respectively.

In this paper, we changed the cost-function to the deep learning form:

11 $$argmin\left({\Vert {D}_{x}{f}_{\theta }\left({\widehat{x}}_{i}\right)-\varphi \Vert }_{2}^{2}+{\lambda }_{1}{\Vert \mathrm{TV}{f}_{\theta }\left({\widehat{x}}_{i}\right)\Vert }_{1}^{1}+{\lambda }_{2}{\Vert M{f}_{\theta }\left({\widehat{x}}_{i}\right)\Vert }_{2}^{2}\right)$$

where ${f}_{\theta }\left({\widehat{x}}_{i}\right)$ denotes the deep learning network outputs. We used the following cost-function for phase retrieval. We used the same parameters to Eq. (10).

### Hardware setup

#### X-ray grating interferometer

We installed two types X-ray TLI setups in a laboratory. In case of the first setup, we used an open-type rotating anode X-ray tube with mean energy 28.0 keV, and an energy-integrating flat-panel type detector with pixel size of 49.6 μm. And all gratings have period of 6.0 μm. The all distance between three gratings were set to 610.0 mm. The second X-ray grating interferometer setup includes a closed-type X-ray tube with mean energy 23.0 keV, and a flat-panel detector which has same specification as the first setup. The G_0_, G_1_, G_2_ gratings have period of 10.0 μm, 3.2 μm, 4.8 μm, respectively. The distance between G_0_ and G_1_ is 300 mm and the distance between G_1_ and G_2_ is 144.0 mm.

#### Neutron grating interferometer

Neutron Talbot–Lau grating interferometer was installed at the cold neutron imaging beam line NG6 at NIST Center for Neutron Research (NCNR)^36^. The wavelength of the source is 0.44 nm with polychromatic beam. The detector is an Andor, a scientific complementary metal-oxide semiconductor (sCMOS) camera which has 50 mm lens, 2160 × 2560 pixels, cooling temperature of -30 °C, and 200 um LiF:ZnS scintillator with the effective pixel size of 51.35 μm (Certain trade names and company products are mentioned in the text or identified in an illustration in order to adequately specify the experimental procedure and equipment used. In no case does such identification imply recommendation or endorsement by the National Institute of Standards and Technology nor does it imply that the products are necessarily the best available for the purpose). The G_0_ and G_1_, G_2_ gratings have same period of 50 μm. The distances between all gratings were set to 4260 mm. Each phase stepping image was obtained for 15 s.

### Dataset preparation

We obtain a set of the phase stepping images to obtain information: phase contrast, transmission, dark-field images. The number of phase stepping can be varied and should be optimized for parameters such as image quality, data acquisition time, noise and so on. Given that we have a certain number of phase stepping, in conventional cases, all phase stepping images are utilized for generating the DPCI, but a DPCI can also be calculated by combination of the phase stepping images. In this way, we can generate a number of DPCIs that have statistical variations, and these images can be used for N2N. In this paper, the transmission and the dark-field images were generated in a conventional way, and the noisy DPCI image pairs were generated by using a combination of the phase stepping images per each iteration and used for training process.

### Data

#### Simulation

We used a numerical phantom with pixel size of 256 × 256 and peppers image with pixel size of 512 × 512. Initially, we padded 32 pixels to the left and right of the images, and normalized to [0, 1]. And we obtained the phase stepping images with Eqs. (1) and (2). We set the phase coefficient ${\theta }_{phantom}=mod\left\{\sqrt{{\left(x/10-20\right)}^{2}+{\left(-y/5+70\right)}^{2}}, 2\pi \right\}$, ${\theta }_{Peppers}=mod\left\{\sqrt{{\left(x/40+10\right)}^{2}+{\left(-y/15+20\right)}^{2}}, 2\pi \right\}$ with horizontal and vertical direction x and y, respectively. And ${\eta }_{k}$ were randomly selected in the range of [-0.05, 0.05], and we set ${a}_{0,ref},{a}_{1,ref}$ to 70,000, 30,000, respectively. The coefficients for the sample images were set using the following method:

12 $${a}_{0,sam}={a}_{0,ref}\times \left(1-{\left(\frac{Image}{4}\right)}^{0.7}\right)$$

13 $${a}_{1,sam}={a}_{1,ref}\times \left(1-{\left(\frac{Image}{1.5}\right)}^{0.5}\right)$$

14 $${\theta }_{sam}=\left(\frac{\partial Image}{\partial x}\right)$$

where *Image* is the normalized images. And we added Poisson noise in the entire of the images. The phase stepping numbers are 8, 12, respectively. We used least-square method for data fitting. The obtained images were randomly flipped for data argumentation.

#### Network training

**Figure 9:**
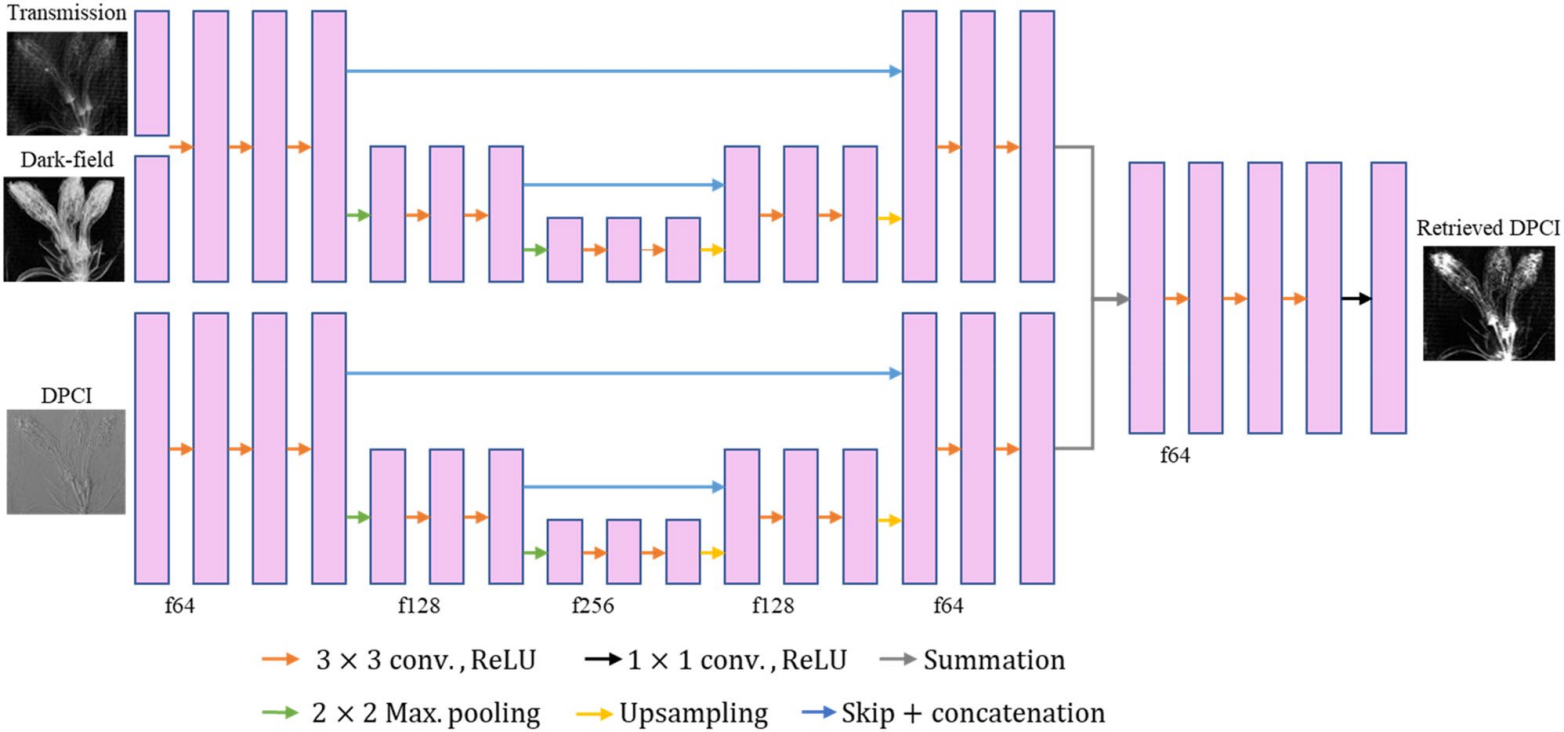
The network architecture used in this study.

We designed a deep learning network in the form of a dual stream network shown in Fig. 9^37^. The dual steam network consists of base/detail layers for denoising. The role of the base layer is to prevent image quality degradation by making base of the result image. In this study, we used the transmission and the dark-field images as the base layer input because they can be obtained using grating interferometer, are not differentiated images, and have a similar shape to the PCI. And we used the DPCI as an input of the detail layer. The role of the detail layer is to obtain detailed features in the result image. Each stream has U-net type network with depth 2, kernel size 3, and rectified linear unit (ReLU)^23^. The features have initially 64 features, and it is doubled as the network is deeper. The features from each stream are added and connected to three convolutional blocks. And the result image can be obtained from the last convolutional layer. The cost function in this study calculates combined losses which are L_2_ loss between differentiated images shown and TV loss, mask image loss shown in Eq. (11). During the training process, the ADAM algorithm was used as an optimizer^38^. The initial learning rate was set to 0.0001 and it decayed $\sqrt{t}$ times with t-th epochs. The network was trained for 100 epochs, and 100 times iterated in one epoch. For every iteration, the reference and the sample images were randomly selected from the half of the total phase stepping number to the total phase stepping number and extracted 64 batches with pixel size of 64 × 64. Network training was performed on a Tensorflow deep learning framework^39^.

### Ethics declaration

The authors declare no humans were directly used in this study.

## Data Availability

The datasets generated during and/or analysed during the current study are available from the corresponding author on reasonable request.
